# Early onset neonatal bloodstream infections in South African hospitals

**DOI:** 10.1186/s12879-024-10406-z

**Published:** 2025-01-20

**Authors:** Genevieve Theron, Adrie Bekker, Larisse Bolton, Andrew Whitelaw, Arnoldus Engelbrecht, Louisa Erasmus, Aaqilah Fataar, Chandre Geldenhuys, Marlize Kunneke, Dave Le Roux, Natasha O’Connell, Kessendri Reddy, Natasha Rhoda, Lloyd Tooke, Mark Wates, Thandi Wessels, Angela Dramowski

**Affiliations:** 1 Department of Paediatrics and Child Health, Faculty of Medicine and Health Sciences, Stellenbosch University, PO Box 241, Cape Town, 8000 South Africa; 2 South African Centre for Epidemiological Modelling and Analysis (SACEMA), School for Data Science and Computational Thinking, Stellenbosch University, Cape Town, South Africa; 3 Division of Medical Microbiology and Immunology, Department of Pathology, Faculty of Medicine and Health Sciences, Stellenbosch University, Cape Town, South Africa; 4 Department of Paediatrics, Worcester Provincial Hospital, Worcester, South Africa; 5 Department of Paediatrics, Paarl Hospital, Paarl, South Africa; 6 Department of Paediatrics, New Somerset Hospital, Cape Town, South Africa; 7 Department of Paediatrics, Khayelitsha District Hospital, Cape Town, South Africa; 8 Department of Neonatology, Cape Town, School of Child and Adolescent Health, Faculty of Health Sciences, Mowbray Maternity Hospital, University of Cape Town, Cape Town, South Africa; 9 Department of Neonatology, Groote Schuur Hospital, Cape Town, South Africa; 10 School of Child and Adolescent Health, Faculty of Health Sciences, University of Cape Town, Cape Town, South Africa; 11 Department of Paediatrics, Karl Bremer Hospital, Cape Town, South Africa

**Keywords:** Neonate, Early onset bloodstream infection, Antimicrobial resistance, Empiric antibiotic, Sepsis

## Abstract

**Background:**

Neonatal sepsis is a leading cause of death in low- and middle- income countries (LMIC). Increasing antibiotic resistance in early onset (< 72 h of life) bloodstream infection (EO-BSI) pathogens in LMIC has reduced the effectiveness of the recommended empiric antibiotic regimen (ampicillin plus gentamicin).

**Methods:**

We retrospectively analysed blood culture-confirmed EO-BSI episodes at nine neonatal units from three central and six peripheral hospitals in the Western Cape Province, South Africa between 1 January 2017 and 31 December 2018. Clinical and electronic laboratory records were reviewed to determine pathogen profile, empiric antibiotic coverage rates and factors associated with EO-BSI attributable mortality, stratified by hospital type.

**Results:**

Of the 8252 blood culture specimens submitted for the investigation of suspected EO-BSI, 136 EO-BSI episodes yielding 141 pathogens were identified with an EO-BSI rate of 1.3 and 0.5 episodes/1000 live births at central and peripheral hospitals respectively. Preterm (93/136; 68.3%) and low birth weight (84/136; 61.8%) neonates were most affected. The predominant pathogens were *Streptococcus agalactiae* (46/136; 34%), *Klebsiella pneumoniae* (17/136; 13%), *Listeria monocytogenes* (11/136; 8%), *Acinetobacter baumannii* (11/136; 8%) and *Escherichia coli* (11/136; 8%). The empiric antibiotic (ampicillin plus gentamicin) coverage rate was 64% (95% CI 51–74) at central hospitals and 84% (95% CI 74–94) at peripheral hospitals. Neonates with Gram-negative EO-BSI and discordant empiric antibiotic therapy had almost four-fold and three-fold higher odds of death respectively.

**Conclusion:**

Preterm and low birth weight neonates are most vulnerable to EO-BSI and have higher odds of death with Gram-negative pathogens and discordant empiric antibiotic therapy.

## Background

The global burden of neonatal infection is substantial, with approximately 1.3 million episodes of severe bacterial infection recorded annually in the first 28 days of life [1]. Low- and middle- income countries (LMIC) are particularly affected, with eight of the 10 countries with the highest neonatal mortality rates worldwide located in Sub-Saharan Africa. Infection is the third most common cause of death in the neonatal period after prematurity and perinatal asphyxia, causing up to 250 000 deaths in sub-Saharan Africa per year [2].

Bloodstream infections (BSI) are the most common infection type encountered in neonates and the most frequently reported in the literature [3]. Early onset BSI (EO-BSI) occur in the first 72 h of life [4, 5] and are traditionally caused by maternal genitourinary tract pathogens such as *Group B streptococcus (GBS)*, *Listeria monocytogenes (L. monocytogenes)* and *Escherichia coli (E. coli)* [5–8]. In LMIC however, Gram-negative and antibiotic-resistant pathogens are increasingly reported as EO-BSI pathogens, in part owing to maternal colonization and suboptimal intrapartum infection prevention practice [6, 9–11].

In South Africa similar findings have been reported with Gram-negative organisms such as *Klebsiella pneumoniae (K. pneumoniae*), *Acinetobacter and E. coli*; predominating in EO-BSI. *GBS* remains a common pathogen in EO-BSI in South Africa [9, 12–14].

For almost five decades, neonates with suspected EO-BSI, have received ampicillin plus gentamicin as the World Health Organization (WHO) recommended first-line empiric antibiotic treatment [15, 16]. In some regions, such as Southeast Asia and Sub-Saharan Africa, there is mounting concern about the continued appropriateness of this regimen owing to high rates of antibiotic resistance [8]. Discordant empiric antibiotic therapy (i.e. bug-drug mismatch) increases the risk of BSI-attributable death three-fold (i.e. death within three calendar days of a positive blood culture) [17].

We investigated the epidemiological profile of EO-BSI and estimated empiric antibiotic coverage rates at nine neonatal units in the Western Cape, South Africa, including three central and six peripheral hospitals.

## Methods

### Study design

A retrospective descriptive analysis of laboratory-confirmed EO-BSI episodes was undertaken including all hospitalised neonates from the nine neonatal units from 1 January 2017 to 31 December 2018 with a culture-confirmed episode of EO-BSI. EO-BSI was defined as a positive blood culture collected in the first three calendar days of life. All neonates whose demographic data, treatment information and clinical outcome could not be determined were excluded. We reviewed several data sources including electronic laboratory results (for full blood count (FBC), C-reactive protein (CRP), blood cultures and antibiotic susceptibility profiles), and clinical records (sex, birth weight, gestational age, and hospital outcome). We report the total number of blood cultures submitted as well as blood culture yield and contamination rates. We compared the demographic profile of neonates with EO-BSI, stratified by hospital type (central versus peripheral) and described factors associated with BSI-attributable mortality. Empiric coverage rates for ampicillin plus gentamicin (with 95% credible intervals) were calculated using a weighted-incidence syndromic combination antibiogram (WISCA) and stratified by hospital type. The Stellenbosch University Health Research Ethics Committee and the Tygerberg Hospital management reviewed and approved the study (N18/07/068) and granted a waiver of individual informed consent. All nine hospitals gave ethical approval.

### Study setting

Nine neonatal units were included from six peripheral (regional and district) hospitals (Helderberg, Paarl, Worcester, Khayelitsha District, Karl Bremer, and New Somerset Hospitals) and three central hospitals (Mowbray Maternity, Tygerberg and Groote Schuur Hospitals) in the Western Cape Province of South Africa. These are the nine largest hospitals in the Western Cape. Central hospitals provided higher level specialised neonatal care, including neonatal intensive care with sub-specialist support, whereas peripheral hospitals (regional and district hospitals) provided neonatal care by general paediatricians, with no or limited neonatal intensive care and no on-site neonatal surgery. Neonates treated at these nine hospitals are either inborn or referred from smaller hospitals or clinics. The neonates in this cohort are representative of all neonates who developed a laboratory-confirmed EO-BSI episode in the Western Cape Province.

### Clinical management of early onset BSI

In these neonatal units, risk factors for EO-BSI that would prompt neonatal evaluation for possible EO-BSI include maternal chorioamnionitis, premature prolonged rupture of membranes (> 18 h) and spontaneous preterm labour. There is no routine screening in pregnant women for Group B streptococcal infection. Clinical signs in the neonate that would prompt infection work-up include apnoea, seizures, temperature instability, respiratory distress, heart rate instability and feeding problems (vomiting, abdominal distention and bloody stools). In neonates with risk factors and/or clinical signs of suspected infection an aseptically collected blood culture specimen (minimum of 1 mL of blood), and a full blood and differential count is submitted to the laboratory at the discretion of the attending clinician. In most neonatal units, a CRP would be performed between 24 and 48 h after the blood culture to inform need for antibiotic continuation. Neonates with suspected EO-BSI are commenced empirically on ampicillin plus gentamicin according to the WHO recommendation.

### Laboratory evaluation of suspected early onset BSI

Central laboratory processing of blood cultures from the nine neonatal units is conducted at Tygerberg and Groote Schuur Hospitals (both large tertiary hospitals) using automated BacT/Alert blood culture incubation systems and BacT/Alert PF Plus bottles (bioMerieux, Marcy l’E`toile, France). For positive blood cultures, a Gram stain is performed from the blood culture broth and the broth is sub-cultured and incubated overnight. Clinicians are telephonically alerted to potential pathogens on Gram stain during working hours. Identification and susceptibility testing is performed using the automated VITEK 2 system (bioMerieux, Marcy l’E`toile, France) and/or disk diffusion testing, interpreted using the annually published Clinical Laboratory Standards Institute (CLSI) breakpoints. Categorisation of likely resistance mechanisms is based on the data generated by the routine phenotypic susceptibility testing.

### Data definitions

A neonate was defined as an infant in their first 28 days of life. EO-BSI was defined as a laboratory-confirmed BSI yielding a known neonatal pathogen and sampled within the first three calendar days of life i.e., a neonate on calendar day 0, 1 or 2 of life. Organisms were classified as pathogens or probable contaminants using the United States Centre for Disease Control (US CDC) commensal list [18]. Coagulase negative staphylococci were deemed to be pathogens if two isolates of the same species were cultured within two calendar days. Duplicate blood culture specimens yielding the same pathogen within 14 days of the original isolate were deleted. The following standard definitions were used to stratify the cohort: low birthweight (< 2500 g), very low birthweight (< 1500 g), extremely low birthweight (< 1000 g), extremely preterm (< 28 weeks), very preterm (28- <32 weeks), moderate to late preterm (32–37 weeks) and term (38 weeks or more). BSI-attributable deaths were defined as death within three calendar days of a positive blood culture [19], whereas BSI-associated death was an in-hospital death occurring four or more calendar days after a positive blood culture. Neonates who died were included in the cohort if they had a laboratory-confirmed EO-BSI episode in the first three calendar days of life.

### Statistical analysis plan

Descriptive analyses were used to compare neonatal demographic characteristics using absolute number, percentage, median and interquartile range (IQR). Continuous data was tested for statistical significance using Mood’s Median Test and categorical data, using Chi-square or Fisher’s exact test. A *p*-value of less than 0.05 was considered statistically significant. Bivariable and multi-variable analyses were carried out to identify factors associated with EO-BSI attributable mortality using generalized linear regression with an assumed error distribution from the binomial family. The relationship between mortality and the associated factors was compared using odds ratios and 95% confidence intervals. Empiric antibiotic coverage rates were reported as point estimates with 95% credible intervals. The data was analysed in R Studio version 2022.07.2 + 57,617 running R statistical software version 4.1.018. The R packages employed during analysis was: tidyverse (1.3.2), lubridate (1.8.0), janitor (2.1.0), openxlsx (4.2.5), RVAidMemoire (0.9–81.2) and broom (1.0.1).

## Results

**Fig. 1:**
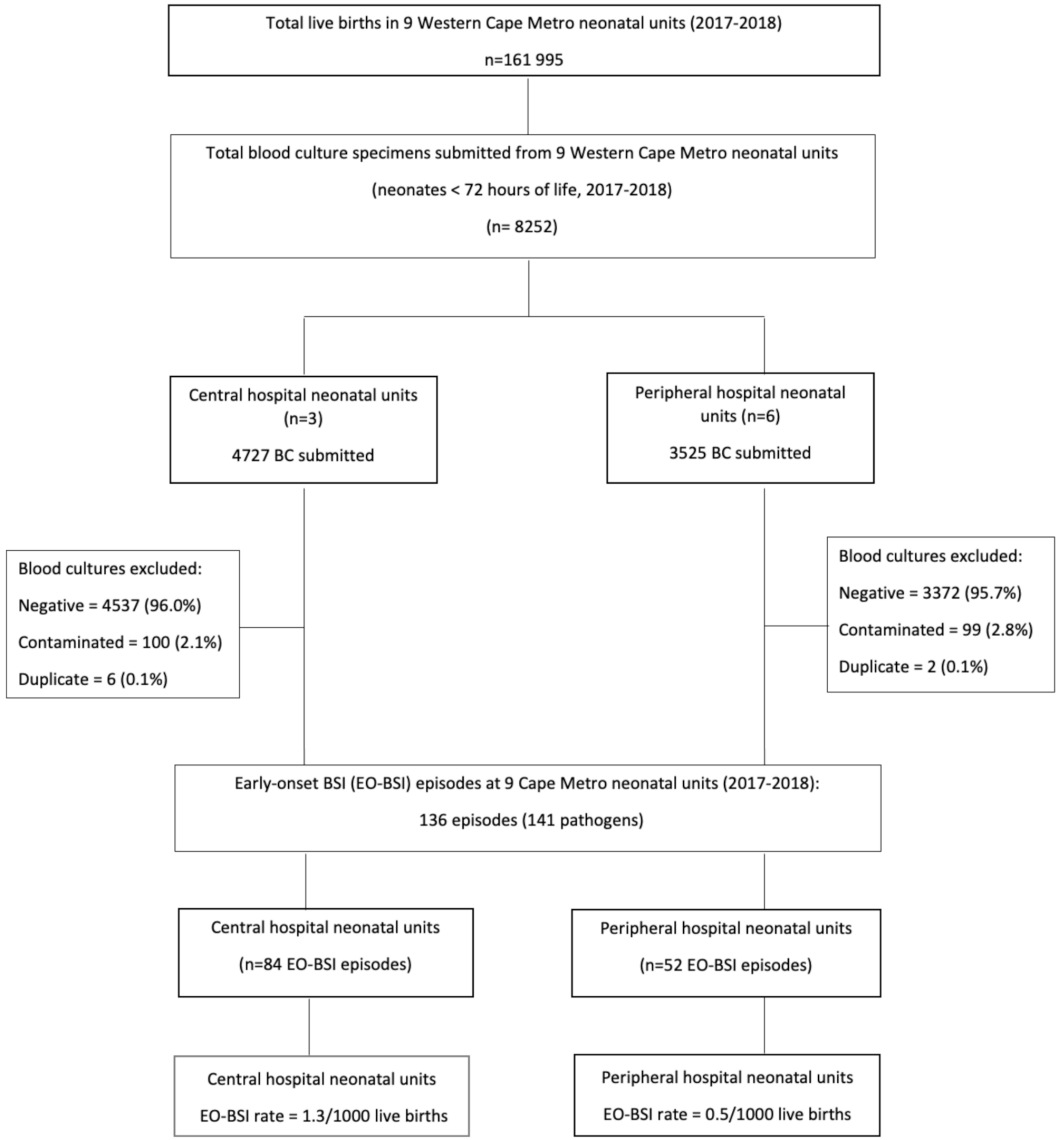
Determination of the study population

A total of 8252 blood culture specimens were submitted for the investigation of suspected EO-BSI at the nine Western Cape neonatal units (2017–2018), with 4727 (57%) from central hospitals and 3525 (43%) from peripheral hospitals (Fig. 1). After excluding negative (95.8%), contaminated (2.4%) and duplicate (0.1%) blood cultures, 136 EO-BSI episodes yielding 141 pathogens were included. There were 84 (61.8%) and 52 (38.2%) EO-BSI episodes at central and peripheral hospitals respectively, with an EO-BSI rate of 1.3 and 0.5 episodes/1000 live births respectively (Fig. 1).

Most EO-BSI episodes affected preterm (93/136; 68.3%) and low birth weight (84/136; 61.8%) neonates (Table 1). Neonates with EO-BSI at peripheral hospitals were more likely to be term (23/52 [44%] vs. 14/84 [17%]; *p* < 0.001) and/or of normal birth weight. About one-third of neonates with EO-BSI at central hospitals required admission to a neonatal intensive care unit. Neonates with EO-BSI at peripheral hospitals had higher rates of HIV exposure (16/52 [31%] vs. 12/84 [14%]; *p* = 0.029). Median length of hospital stay for all neonates with EO-BSI was 12 (IQR 6–30) days.

**Table 1:** Characteristics of neonates with early-onset bloodstream infection (EO-BSI)

Neonatal demographics	Total	Central hospitals	Peripheral hospitals	*P*-value
Total EONS episodes	136	84	52	-
Gestational age at birth (weeks), *n* (%)				
<28 weeks	12 (9%)	12 (14%)	0 (0%)	
28 - <32 weeks	35 (26%)	26 (31%)	9 (17%)	< 0.001
32–37 weeks	46 (34%)	26 (31%)	20 (39%)	
≥ 38 weeks	37 (27%)	14 (17%)	23 (44%)	
unknown	6 (4%)	6 (7%)	0 (0%)	
Birth weight (grams), *n* (%)				
< 1000 g	21 (16%)	20 (24%)	1 (2%)	
1000–1499 g	26 (19%)	19 (23%)	7 (13%)	
1500–2500 g	37 (27%)	21 (25%)	16 (31%)	< 0.001
> 2500 g	44 (32%)	18 (21%)	26 (50%)	
unknown	8 (6%)	6 (7%)	2 (4%)	
Sex, *n* (%)				
male	60 (44%)	36 (43%)	24 (46%)	
female	76 (56%)	48 (57%)	28 (54%)	0.726
Maternal HIV status, *n* (%)				
positive	28 (21%)	12 (14%)	16 (31%)	
negative	108 (79%)	72 (86%)	36 (69%)	0.029
Ward type, *n* (%)				
Intensive care/high care unit	26 (19%)	25 (30%)	1 (2%)	< 0.001
Nursery/ward	110 (81%)	59 (70%)	51 (98%)	
Number of organisms in the blood culture, *n* (%)				
monomicrobial	131 (96%)	81 (96%)	50 (96%)	1
polymicrobial	5 (4%)	3 (4%)	2 (4%)	
Length of hospital stay (median, IQR)	12 (6–30)	16.5 (4-38.3)	12 (7 -16.3)	0.382
Laboratory indices at infection onset (median, IQR)^*^				
C-reactive protein (mg/L)	26 (11–76)	30.5 (11.3–76)	18.5 (11–69.5)	0.426
White cell count (x 10⁹/L)	7.5 (3.6–13.4)	7.2 (2.7–12)	7.5 (4.2–13.4)	0.849
Haemoglobin (g/Dl)	15.6 (13.5–17.1)	14.9 (12.7–16.4)	16.7 (15–18.2)	0.004
Platelet count (x10⁹/L)	187 (120–227)	167 (97–210)	205 (161–243)	0.079
Mortality, *n* (%)				
crude mortality	38 (28%)	29 (35%)	9 (17%)	0.032
sepsis-attributable	28 (74%)	21 (72%)	7 (78%)	0.129
sepsis-associated	10 (26%)	8 (28%)	2 (22%)	0.317

^*^Of the 136 EONS episodes, 24 (18%) C-reactive protein (CRP), 23 (17%) White cell count (WCC), 26 (19%) Platelets (PLC) and 23 (17%) Haemoglobin (Hb) entries were missing

**Fig. 2:**
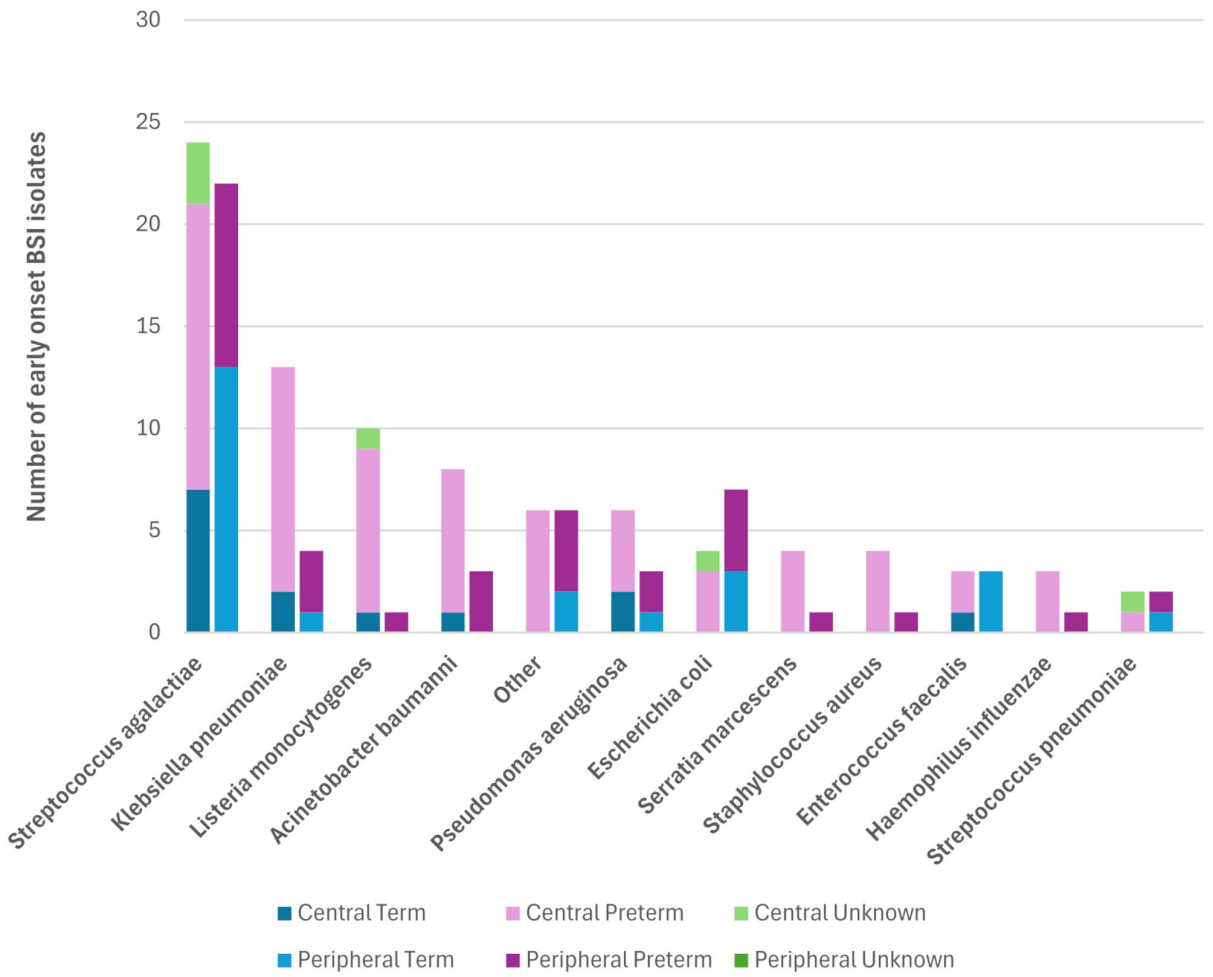
Spectrum of early onset BSI pathogens at nine neonatal units in the Western Cape Province, South Africa EO-BSI: early onset blood stream infection Other: *Enterobacter cloacae (1)*, *Aeromonas hydrophila (1)*, *Neisseria elongata (1)*, *Citrobacter species (1)*, *Enterococcus faecium (2)*, *Acinetobacter ursingii (1)*, *Burkholderia gladioli (1)*, *Sphingomonas paucimobilis (2)*, *Pseudomonas orizihabitans (1)*, *Proteus mirabilis (1)*

Figure 2. illustrates the pathogen spectrum reported at both central and peripheral hospitals; 4% of EO-BSI episodes were polymicrobial. There was a slight predominance in Gram-positive pathogens (74/136; 54%) in comparison to Gram-negative pathogens (67/136; 49%) with no fungal pathogens being cultured. For single organism blood cultures, the predominant EO-BSI pathogens were GBS (46/136; 34%), *K. pneumoniae* (17/136; 13%), *L. monocytogenes*, *Acinetobacter baumannii (A. baumannii)* and *E. coli* (11/136; 8% each). Term neonates (*n* = 37) isolated 12 Gram-negative pathogens (32.4%) and 26 Gram-positive pathogens (70.3%) respectively (one polymicrobial EO-BSI episode). Preterm neonates (*n* = 93) had a higher proportion of EO-BSI caused by Gram-negative pathogens (58.1%) than term neonates, with 43 Gram-positive pathogens (46.2%) (including four polymicrobial EO-BSI episodes). There was one Gram-negative pathogen and five Gram-positive pathogens for which the neonate’s gestational age was unknown.

**Fig. 3:**
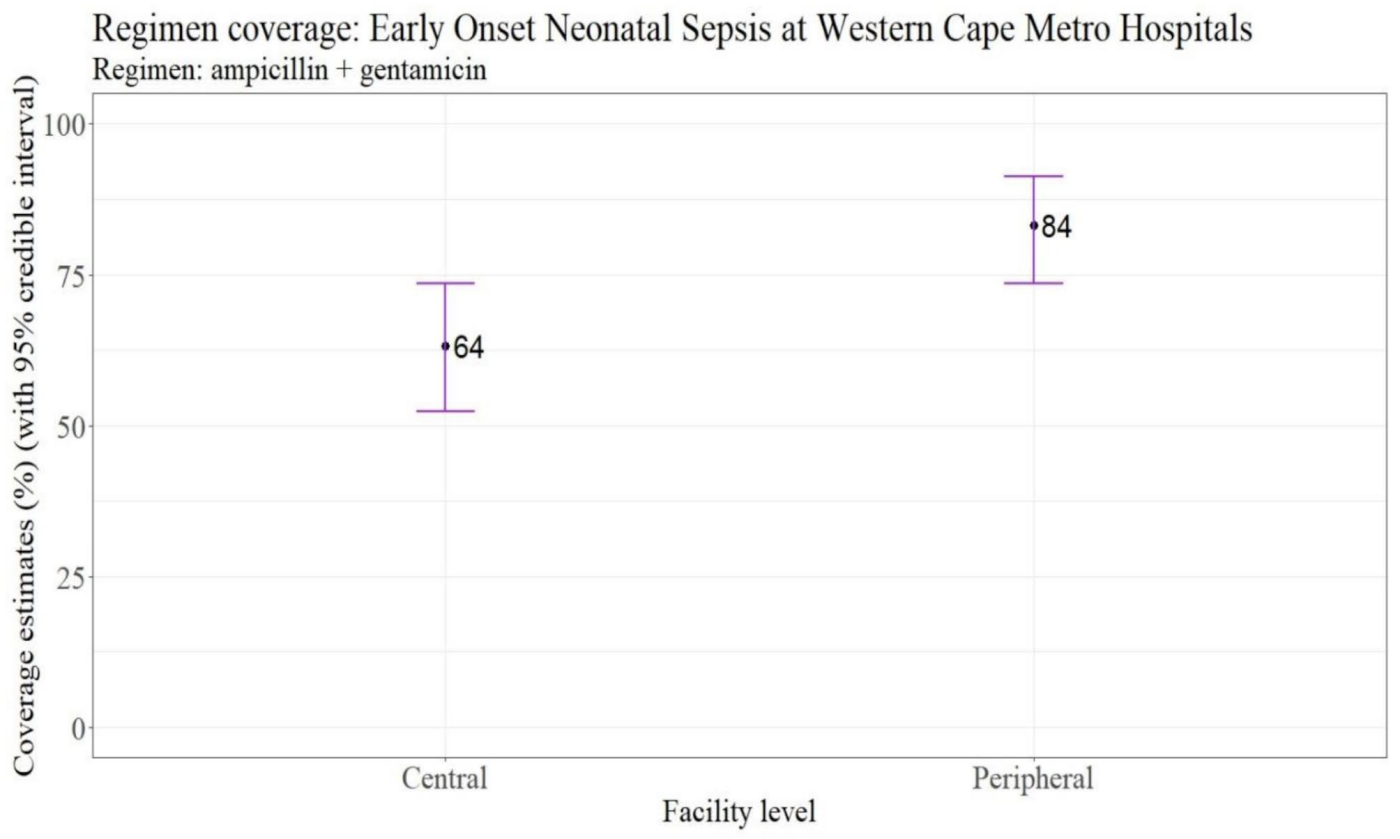
Empiric antibiotic coverage rate for early onset BSI Empiric antibiotic (ampicillin + gentamicin) coverage point estimate (with 95% credible interval) for early onset bloodstream infection pathogens calculated by weighted-incidence syndromic antibiogram (WISCA) for nine Western Cape hospital neonatal units, stratified by site (central versus peripheral hospital). The WISCA was derived using 117 of the 141 (83%) pathogens isolated for EO-BSI. In calculating the coverage estimates, the model assumes that neonates with suspected EO-BSI received the World Health Organization recommended empiric therapy (ampicillin plus gentamicin)

The empiric antibiotic coverage rate for ampicillin plus gentamicin in EO-BSI was 64% (95% CI 51–74) at central hospitals and 84% (95% CI 74–94) at peripheral hospitals (Fig. 3). Among the 27 EO-BSI episodes with non-susceptibility to empiric antibiotic therapy (ampicillin plus gentamicin), *K pneumoniae* predominated (11; 41%) followed by *A. baumannii* (7; 26%), *Haemophilus influenzae (H. influenzae)* (4; 15%), *Staphylococcus aureus (S. aureus)* (2; 7%), *Enterococcus faecium (E. faecium)* (2; 7%) and *Burkholderia gladioli (B. gladioli)* (1; 4%).

Almost one-third of neonates with EO-BSI died, with higher crude mortality rates at central compared to peripheral hospitals (29/84 [35%] vs. 9/52 [17%]; *p* = 0.032). Of the 38 neonates who died, 28 deaths (74%) were sepsis-attributable and 10 (26%) were sepsis-associated. The highest crude mortality rates by EO-BSI pathogen were observed for *S. agalactiae* (8/35, 23%), *K. pneumoniae* (8/35, 23%), *A. baumannii* (6/35, 17%) and *Pseudomonas aeruginosa (P. aeruginosa)* (5/35, 14%).

Factors significantly associated with BSI-attributable mortality on multivariate analysis were very preterm (OR 11.4; 95% CI 3.2–54.5) and extremely low birth weight neonates (< 1000 g) (OR 14.3; 95% CI 3.7–65.6) (Table 2). Neonates with Gram-negative blood stream infection had almost four-fold higher odds of death compared to neonates with Gram-positive BSI. Neonates with discordant empiric antibiotic therapy (bug-drug mismatch) had almost three-fold higher odds of death compared to neonates who received concordant empiric therapy.

**Table 2:** Factors associated with early onset BSI-attributable mortality (*n* = 31)

Variable	Univariable analysis		Multi-variable analysis
	*p*-value	Odds ratio (95% confidence interval)	Odds ratio (95% confidence interval)
Gender			
Female		Reference	
Male	0.596	1.26 (0.53–2.93)	
Birth weight category			
Normal birth weight		Reference	
Extremely LBW	< 0.001	14.29 (3.74–65.59)	7.19 (1.52–39.5)
Very LBW	0.088	3.33 (0.85–14.45)	2.61 (0.64–11.8)
LBW	0.143	2.69 (0.74–11.15)	2.17 (0.57–9.28)
Main pathogen type			
Gram-positive		Reference	
Gram-negative	0.004	3.78 (1.56–9.92)	2.38 (0.83–7.14)
Level of care			
Peripheral		Reference	
Central	0.077	2.35 (0.95–6.42)	1.62 (0.56–5.01)
Maternal HIV status			
Negative		Reference	
Positive	0.681	0.80 (0.25–2.22)	
Empiric antibiotic concordance‡			
Concordant		Reference	
Discordant	0.025	2.93 (1.12–7.50)	1.21 (0.37–3.75)

## Discussion

We described the demographic and clinical profile of neonates treated for EO-BSI at nine South African hospitals; preterm and low birth weight neonates comprised two-thirds of the cohort. EO-BSI rates were comparable to that reported in the international literature, but pathogen spectrum had a higher proportion of Gram-negative pathogens than that of high-income country neonatal EO-BSI [20]. Leading EO-BSI pathogens were *S. agalactiae*, *L. monocytogenes*, *K. pneumoniae E. coli and A. baumannii*. Ampicillin plus gentamicin provided adequate coverage for 64% and 84% of EO-BSI episodes at central and peripheral hospitals respectively. There was a predominance of Gram-negative BSI in preterm babies whereas term babies were more likely to have a Gram-positive BSI. Neonates with Gram-negative EO-BSI and discordant empiric antibiotic therapy had three-fold higher odds of death.

### EO-BSI rates

The incidence rate of EO-BSI worldwide varies from 1.0 to 4.6 per 1000 live births but is highest in VLBW neonates at 11–36 per 1000 live births [5]. We determined an EO-BSI rate of 1.3 and 0.5 episodes/1000 live births at central and peripheral hospitals respectively, with an overall rate of 0.8/1000 live births.

### Demographic and clinical profile

Low birth weight and prematurity has previously been associated with EO-BSI [6] and was similarly demonstrated in this neonatal cohort with EO-BSI (69% preterm and 62% LBW). Most EO-BSI episodes occurred at the three central hospitals where most women with high-risk pregnancies are delivered. The proportion of neonates with EO-BSI born to women living with HIV was marginally higher than the background maternal HIV prevalence in the province (21% versus 18%) [21]. The higher antenatal HIV prevalence observed in peripheral hospitals is likely due to the higher HIV prevalence in the sub-districts served by these hospitals e.g. antenatal HIV prevalence in Khayelitsha was 33% in 2015 [22].

### Pathogen profile

The three most common EO-BSI pathogens identified were *S. agalactiae*, *K. pneumoniae* and *L. monocytogenes.* Maternal genitourinary and gastrointestinal tract colonisation is a well-recognised risk factor for neonatal GBS disease together with chorioamnionitis and prolonged rupture of membranes, although we were not able to document these owing to the retrospective study design [23–25]. The high prevalence of *L. monocytogenes* EO-BSI observed in this cohort is likely due to the nationwide Listeria outbreak that occurred in 2017 and 2018 [26]. *K. pneumoniae* is the leading neonatal BSI pathogen globally, especially in healthcare-associated infections, however, multi-drug resistant Klebsiella is increasingly reported as an EO-BSI pathogen in LMIC [27]. *E. coli* is a frequent EO-BSI pathogen [27], and in this cohort was the third most common pathogen along with *A. baumannii*, which is typically isolated in healthcare-associated infections, suggesting lapses in infection control with early neonatal colonization and subsequent bloodstream invasion.

### Antibiotic coverage

The WHO-recommended first-line antibiotic regimen for treatment of EO-BSI remains ampicillin and gentamicin [15, 16], but several studies from LMICs have reported increasing pathogens resistance to these agents [6]. In this cohort, estimated antibiotic coverage rates for the currently recommended regimen (ampicillin plus gentamicin) were sub-optimal at central hospitals (64%) but retained acceptable activity against EO-BSI pathogens at peripheral hospitals (84%). A possible reason for this could be the higher incidence of extremely low birth weight/preterm neonates at central hospitals, who have longer hospital stays, greater use of indwelling devices and surgical comordities, which predispose to higher rates of Gram-negative BSI. These findings are concerning and imply increasing vertical (mother to baby) transmission of multi-drug resistant pathogens and/or increased early life horizontal transmission of antibiotic-resistant pathogens from the maternity and neonatal unit environment. The data highlights the need to conduct regular reviews of antibiotic coverage at local institutional, provincial, and national level to inform empiric treatment recommendations for neonatal sepsis (both EO-BSI and healthcare-associated BSI). Antimicrobial stewardship programmes and infection prevention control measures are also key in preventing infection transmission and optimising antibiotic use [2].

### Factors associated with EO-BSI attributable mortality

In this cohort, very preterm and extremely low birth weight neonates were 11 times and 14 times more likely to die compared to term neonates, independent of hospital type. Other studies have also reported an inverse relationship between gestational age/ birth weight and EO-BSI mortality [28, 29]. More deaths occurred at central versus peripheral hospitals indicating the higher burden and severity of disease at central hospitals where very preterm and medically complex neonates are cared for.

Mortality from Gram-negative BSI pathogens was almost four times higher than mortality associated with Gram-positive EO-BSI. This is in keeping with other studies in LMIC settings where Gram-negative pathogens predominate as causes of EO-BSI [4, 15]. Death attributable to EO-BSI was three-fold more likely in neonates with discordant empiric antibiotic therapy. This is in keeping with a multi-centre retrospective study of neonatal and paediatric BSI that found a 3-fold increase 30-day mortality rates with discordant empiric antibiotic treatment [17].

### Limitations and strengths

This cohort included only blood culture-confirmed cases of EO-BSI, which substantially underestimates the true burden of neonates investigated and treated for suspected sepsis. The rate of sepsis in very preterm neonates was not calculated as the total number of patients with and without sepsis was not available. Owing to the retrospective design, only in-vitro antibiotic susceptibility to ampicillin plus gentamicin could be calculated as prescription charts were not available to review. There was very limited access to maternal demographic data, including antibiotic use, duration of rupture of membranes and antiretroviral treatment for mothers living with HIV infection. The study included the nine largest neonatal units in the Western Cape Province, but the findings may not be generalisable to all South African neonatal units owing to regional variability in spectrum of EO-BSI pathogens. This study is one of the first to employ use of weighted-incidence syndromic antibiograms to guide empiric antibiotic recommendations for neonatal sepsis in South Africa.

## Conclusion

Neonates with Gram-negative EO-BSI and discordant empiric antibiotic therapy had increased odds of infection-attributable death. Empiric antibiotic recommendations for EO-BSI at central hospitals should be reconsidered given the low coverage rates observed. Regular review of antibiotic coverage rates is a critical activity in LMIC neonatal units to ensure ongoing effectiveness of empiric therapy for EO-BSI.

## Data Availability

The datasets generated during and/or analysed during the current study are available from the corresponding author on reasonable request and completion of a data sharing agreement.

## Abbreviations

AMR: Antimicrobial resistant
BSI: Bloodstream infections
CLSI: Clinical Laboratory Standards Institute
CRP: C-reactive protein
EO-BSI: Early onset bloodstream infections
FBC: Full blood count
GBS: Group B Streptococcus
LMIC: Low- and middle-income countries
NHLS: National Health Laboratory Service
OR: Odds ratio
US CDC: United States Centre for Disease Control
WISCA: Weighted-incidence syndromic combination antibiogram
WHO: World Health Organization
